# Revisiting the Role of Lorazepam as an Adjunct in the Management of Chemotherapy-Induced Nausea and Vomiting: A Systematic Review and Meta-Analysis

**DOI:** 10.3390/biomedicines14040924

**Published:** 2026-04-17

**Authors:** Tzu-Rong Peng, Hung-Hong Lin, Ta-Wei Wu, Chin-Yu Hsu

**Affiliations:** 1 Department of Pharmacy, Taipei Tzu Chi Hospital, Buddhist Tzu Chi Medical Foundation, New Taipei City 23142, Taiwan; tzu.rong@tzuchi.com.tw (T.-R.P.); tawei@tzuchi.com.tw (T.-W.W.); 2 School of Pharmacy, College of Pharmacy, Taipei Medical University, Taipei 11031, Taiwan; 3 Department of Nursing, Tzu Chi University, Hualien 970374, Taiwan; 4 Department of Pharmacy, Chia-Nan University of Pharmacy and Science, Tainan 717301, Taiwan; h120076@ms13.hinet.net; 5 Department of Pharmacy and Master Program, Tajen University, Pingtung 90741, Taiwan; 6 Department of Pharmacy, En Chu Kong Hospital, New Taipei City 23702, Taiwan

**Keywords:** lorazepam, chemotherapy-induced nausea and vomiting, antiemetic therapy, meta-analysis, supportive care

## Abstract

**Background/Objectives**: While standard antiemetic regimens have evolved, breakthrough symptoms and anticipatory nausea persist. Lorazepam has historically been used as an adjunct, yet a comprehensive re-evaluation of its efficacy across historical trials is lacking. This study provides a synthesis of clinical evidence to re-evaluate the adjunctive therapeutic value of lorazepam, potentially addressing persistent gaps in emesis control, such as anticipatory and refractory symptoms. **Methods**: Following PRISMA guidelines, we analyzed eight randomized controlled trials (*n* = 864) published between 1989 and 1997. Primary endpoints included complete and improved responses for emesis and nausea. **Results**: Eight trials (*n* = 864), published between 1989 and 1997, met the inclusion criteria. Lorazepam-containing regimens significantly increased the complete response rate for overall emesis (OR = 1.55; 95% CI, 1.12–2.14; *p* = 0.008) and improved the response rate (OR = 1.50; 95% CI, 1.03–2.19; *p* = 0.04). Subgroup analysis of acute emesis showed consistent benefits (complete response OR = 1.77; 95% CI 1.23–2.55; *p* = 0.002). Nausea control also favored lorazepam, although the differences were not statistically significant. Sedation was more frequent with lorazepam (RR = 2.67; 95% CI 1.54–4.63), although no serious adverse events were reported. **Conclusions**: By revisiting decades of clinical evidence, this meta-analysis confirms that lorazepam provides a significant therapeutic advantage in controlling chemotherapy-related vomiting, particularly during the acute phase. While its direct efficacy alongside modern agents like NK1 receptor antagonists remains to be fully elucidated, the anxiolytic and amnestic properties of lorazepam remain a potential adjunct for managing complex CINV profiles. Careful dose titration is necessary to balance efficacy with sedation.

## 1. Introduction

Cancer is among the most prevalent and life-threatening diseases worldwide, with steadily increasing incidence and mortality rates [1]. Advances in systemic therapy, particularly the introduction of cytotoxic chemotherapy, have contributed to prolonged survival in patients with many cancer types [2]. However, chemotherapy is frequently associated with significant treatment-related toxicities, of which chemotherapy-induced nausea and vomiting (CINV) is a substantial adverse effect [3]. CINV not only impairs nutritional status and functional well-being but also disrupts treatment adherence, ultimately diminishing the overall quality of life (QOL) of patients with cancer [4,5]. Surveys have consistently demonstrated that nausea and vomiting are among the most severe and intolerable complications of chemotherapy, underscoring the importance of effective supportive care strategies [6]. Moreover, modern prospective data indicate that psychological distress, such as anxiety regarding physical appearance and emotional problems, significantly exacerbates the emetic experience [7].

Current antiemetic protocols typically involve combinations of 5-hydroxytryptamine type 3 (5-HT3) receptor antagonists, neurokinin-1 receptor antagonists, dopamine antagonists, and corticosteroids. Although these regimens have markedly reduced the incidence of acute and delayed CINV, breakthrough symptoms continue to occur in several patients. Moreover, anticipatory nausea and vomiting, which are often triggered by previous negative treatment experiences and anxiety, remain particularly resistant to standard pharmacological interventions. These limitations underscore the need to investigate adjunctive therapies that target both the physiological and psychological aspects of CINV [8].

Psychosocial distress, particularly anxiety, is a significant risk factor for anticipatory and breakthrough CINV. Patients who experience heightened anxiety before chemotherapy sessions are more likely to develop nausea and vomiting even without chemotherapy exposure. The interplay between psychological state and physical symptoms suggests that integrating anxiolytic strategies into antiemetic regimens could provide additional benefits beyond conventional pharmacotherapy [9].

Benzodiazepines, particularly lorazepam, are widely used for the short-term management of anxiety, agitation, and sleep disturbance. Lorazepam potentiates gamma-aminobutyric acid (GABA) neurotransmission, producing anxiolytic, sedative, and amnestic effects. These properties make lorazepam an attractive candidate for managing anticipatory CINV, as it may reduce conditioned responses and alleviate the anxiety associated with chemotherapy. Additionally, its sedative and amnestic effects contribute to improved treatment tolerance. Significantly, lorazepam is less frequently associated with extrapyramidal adverse effects, making it a potentially safe adjunctive agent [10,11,12,13].

Over the past several decades, lorazepam has been evaluated in various clinical settings as a part of combination antiemetic regimens, often alongside agents such as metoclopramide, corticosteroids, or 5-HT3 receptor antagonists. Some studies have reported favorable outcomes, including reductions in the frequency and severity of nausea and vomiting as well as improvements in patient comfort and quality of life [7,14]. However, other studies have reported modest or inconsistent results. Given these mixed findings and the lack of a comprehensive synthesis of available evidence, the precise role of lorazepam in preventing and managing CINV remains uncertain.

A randomized, double-blind, crossover study by Bishop et al. in 1984 compared lorazepam with a placebo in patients receiving cytotoxic chemotherapy and prochlorperazine [15]. Lorazepam significantly reduced the severity and duration of nausea, as well as the severity of vomiting and the number of vomiting episodes, compared to the placebo. Anxiety was reduced during lorazepam courses, but not significantly when compared with the placebo. Sedation induced by lorazepam was also considerably higher. Overall, patients preferred lorazepam courses, although this preference was significant only in the subset of patients who received doxorubicin and cyclophosphamide.

In another study by Bishop et al. in 1992 [16], the addition of dexamethasone to prochlorperazine and lorazepam significantly improved patients’ experiences while receiving chemotherapy. This combination regimen was more effective than either prochlorperazine or lorazepam alone in controlling nausea and vomiting. Currently, the 2020 ASCO guideline on antiemetics also mentioned that lorazepam is a useful adjunct to antiemetic drugs for CINV and breakthrough nausea and vomiting, although this recommendation is based on low-quality evidence or informal consensus [17]. A 2024 retrospective analysis of real-world data from China showed that lorazepam is beneficial for antiemesis, particularly for anticipatory nausea and vomiting [14]. However, a systematic review by Grunberg et al. in 2007 [18] has indicated lorazepam as the standard of care for CINV in some settings; however, its role may vary depending on specific patient factors and institutional practices.

Despite the clinical success of modern triplets or quadruplet antiemetic regimens (e.g., NK1 receptor antagonists and olanzapine), breakthrough and anticipatory symptoms remain significant clinical challenges, affecting up to 30–40% of patients [7]. While contemporary agents target specific neurotransmitter pathways (serotonin, substance P, dopamine), they may not fully address the psychological and anxiety-driven components that exacerbate emesis [19]. Therefore, a systematic re-evaluation of lorazepam—a drug with unique anxiolytic and amnestic properties—is necessary to define its complementary role in modern, multidimensional CINV management. By synthesizing evidence across historical trials, this study aims to provide a robust baseline for integrating anxiolytic adjuncts into current practice.

Given the variability in historical study outcomes and the ongoing challenges in modern emesis control, further research is required to define the definitive role of lorazepam. Hence, this systematic review and meta-analysis aimed to (1) evaluate the efficacy of lorazepam in reducing CINV incidence and severity, (2) examine its specific role in anticipatory symptoms, (3) assess its impact on quality of life, and (4) summarize the safety considerations associated with its use.

## 2. Materials and Methods

This systematic review and meta-analysis were conducted in accordance with the Preferred Reporting Items for Systematic Reviews and Meta-Analyses (PRISMA) guidelines, Table S1 [20]. Trials were included if they met the following criteria: (1) had a randomized controlled trial (RCT) design; (2) evaluated a lorazepam-containing regimen for CINV prevention using a comparator group; and (3) provided clear definitions of drug types and study outcomes. The criteria for study exclusion were as follows: (1) unclear reporting of drug types, (2) unclear or non-RCT study designs, or (3) interventions not based on lorazepam-containing regimens for the prevention of CINV or the absence of a comparator group. This study was prospectively registered with PROSPERO (registration number: CRD420251145888).

### 2.1. Search Strategy

Studies were identified through computerized searches of the PubMed, Embase (Ovid), and Cochrane Library databases. The following terms were used as medical subject headings and combined in a Boolean search format: “lorazepam,” “ativan,” “chemotherapy-induced nausea and vomiting (CINV),” “nausea,” vomiting,” “chemotherapy-related nausea and vomiting,” and “acute phase.” Additional terms related to the study design, such as “randomized controlled trial,” “controlled clinical trials,” “randomized,” “random,” “placebo,” and “trial,” were also included. The “Similar Articles” section in PubMed was used to broaden the search. All abstracts, studies, and citations retrieved after the last search on 25 August 2025 were reviewed. Language restrictions were not imposed. Interventionary studies involving animals or humans, and other studies that require ethical approval, must list the authority that provided approval and the corresponding ethical approval code.

### 2.2. Data Extraction

Two reviewers (T.-R.P. and H.-H.L.) independently extracted data from the included studies using a standardized form. The extracted information included study characteristics such as author, year of publication, study design, and sample size; treatment arms and interventions including lorazepam-containing regimens; whether outcomes were evaluated in the acute or delayed emesis phase; patient demographics including age, sex, and cancer type; chemotherapy details including regimen type and treatment duration; and clinical outcomes such as complete response, complete and improved control of acute emesis, overall emesis control, nausea control, and safety outcomes, with particular attention to sedation. Data extracted by the two reviewers were compared for consistency, and any discrepancies were resolved through discussion; a third expert adjudicated unresolved disagreements.

### 2.3. Methodological Quality Appraisal

Two reviewers (T.-R.P. and H.-H.L.) independently assessed the methodological quality of each included study using version 2 of the Cochrane Risk of Bias Tool for Randomized Trials (RoB 2), as outlined in the Cochrane Handbook for Systematic Reviews of Interventions. The assessment considered the following domains of potential bias: bias arising from the randomization process, bias due to deviations from the intended interventions, bias due to missing outcome data, bias in outcome measurement, and bias in the selection of reported results.

### 2.4. Outcome Assessments

The efficacy of the lorazepam-based prophylactic regimens was evaluated using an outcome assessment. The primary outcomes were complete response and improved response (full and partial controls). The other outcomes included nausea, vomiting, and adverse events. Additionally, a subgroup analysis was conducted to evaluate the effect of lorazepam-based prophylaxis on complete and improved responses (complete and partial control) to acute emesis.

### 2.5. Statistical Analysis

Statistical analyses were conducted using the Review Manager (version 5.4; Cochrane Collaboration, Oxford, UK) and R software (version 4.4.3) following the PRISMA guidelines. Dichotomous outcomes were summarized using weighted odds ratios (ORs) with 95% confidence intervals (CIs) to determine the precision of effect estimates. Pooled ORs were calculated using the DerSimonian and Laird random-effects model, and data were combined only when the studies had sufficiently similar clinical and methodological characteristics. Heterogeneity among the studies was assessed using the I^2^ statistic, which quantifies the proportion of total variability attributable to between-study differences. In studies that evaluated multiple dosages, a dosage consistent with that used in the other included studies was selected for meta-analysis.

## 3. Results

### 3.1. Literature Search

**Figure 1:**
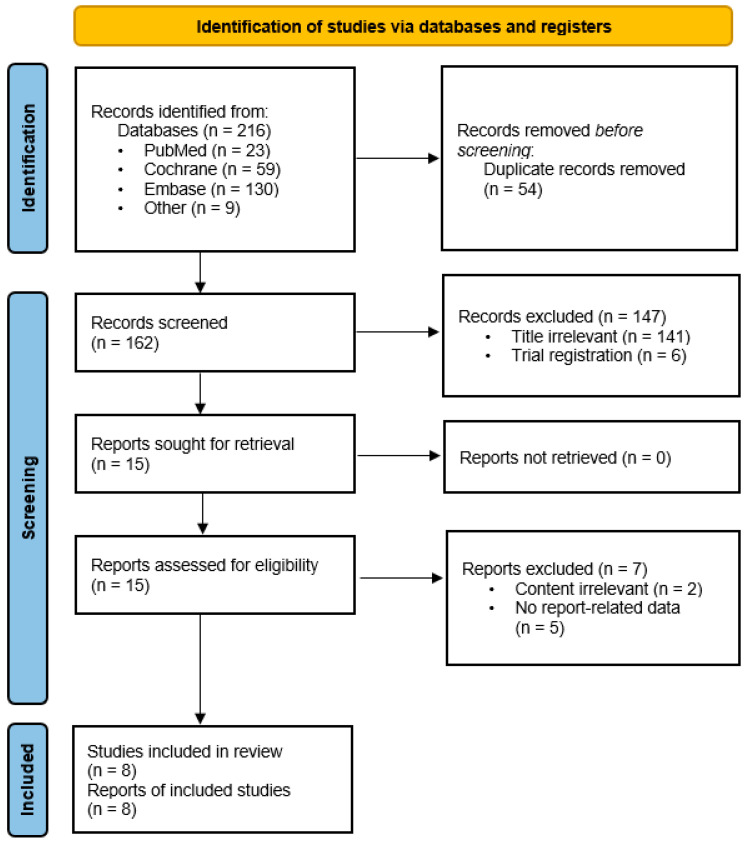
Preferred Reporting Items for Systematic Reviews and Meta-Analyses flow diagram for study selection.

A total of 216 studies were identified through database searches, including PubMed (*n* = 23), Cochrane Library (*n* = 59), Embase (OVID) (*n* = 130), and other sources (*n* = 9). After removing 54 duplicates, 162 records were screened based on title and abstract. Of these, 147 were excluded (141 due to the irrelevance of the title and six due to being trial registrations), leaving 15 articles for a full-text review. Following the eligibility assessment, seven reports were excluded (two due to irrelevant content and five with no report-related data). Finally, eight studies met the inclusion criteria and were included in this systematic review (Figure 1). The detailed search strategies are presented in Appendix A.

### 3.2. Study Characteristics

**Figure 2:**
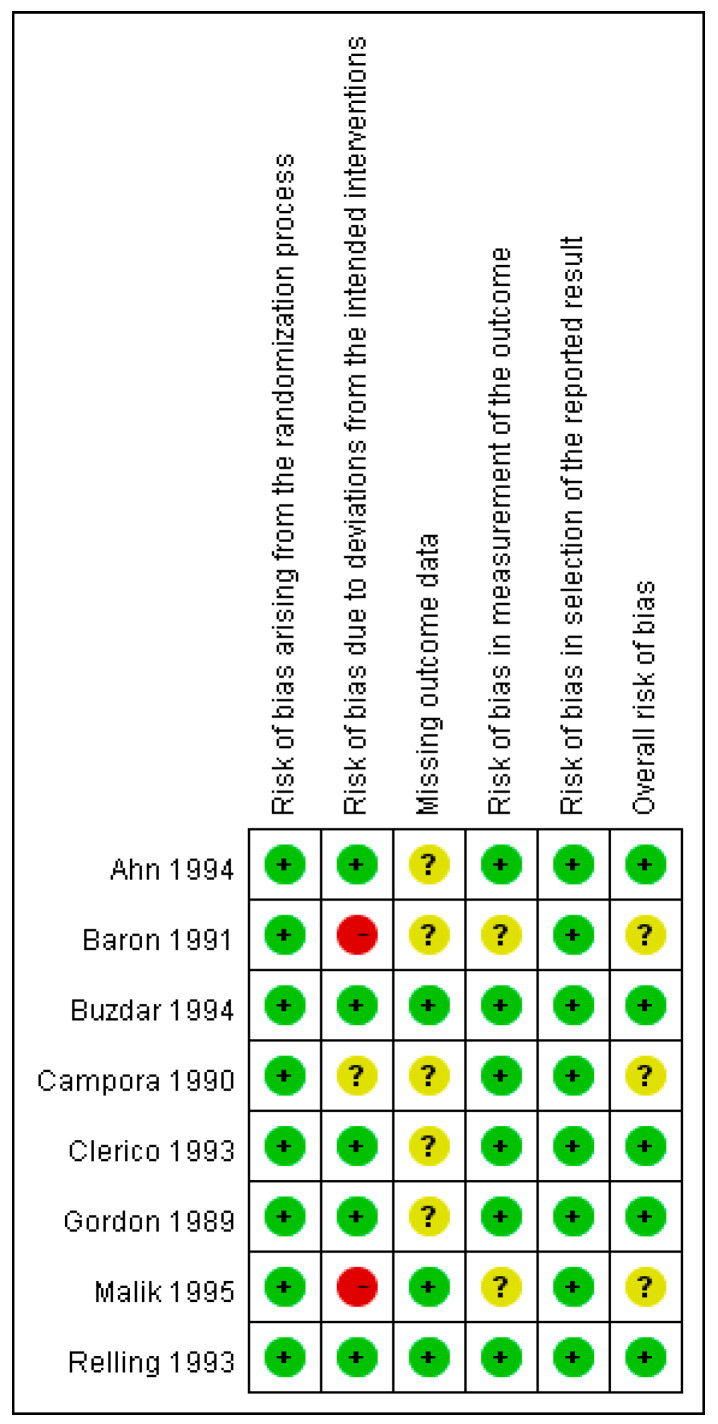
Risk-of-bias assessments for randomized clinical trials were included in the meta-analysis [11,21,22,23,24,25,26,27].

Eight randomized or prospective trials published between 1989 and 1997 with approximately 864 participants (sample sizes ranging from 53 to 282) were included. Most studies were double-blind RCTs, and only one was a prospective randomized trial. Chemotherapy regimens included cisplatin-based therapy as well as other commonly used agents, such as cyclophosphamide, doxorubicin, fluorouracil, vincristine, dacarbazine, carmustine, and methotrexate. Seven studies used a two-arm design, whereas Ahn employed a three-arm design. The interventions consisted of lorazepam in combination with standard antiemetics, such as ondansetron, dexamethasone, metoclopramide, methylprednisolone, and promethazine, compared with regimens that did not contain lorazepam. The primary outcomes included acute emesis, delayed emesis, or both. Most trials have evaluated the effect of lorazepam on delayed emesis, and several have also reported outcomes for acute emesis. The key characteristics of the included studies are summarized in Table 1, and the risk-of-bias (ROB) assessment results are shown in Figure 2.

RCTs evaluating lorazepam-containing regimens for CINV prevention were included. All studies compared lorazepam-containing antiemetic regimens with standard regimens that did not contain lorazepam. Most trials enrolled adult patients receiving moderately to highly emetogenic chemotherapy and lorazepam combined with other standard antiemetics, such as 5-HT receptor antagonists, dopamine antagonists, or corticosteroids.

### 3.3. Overall Emesis Control

**Figure 3:**
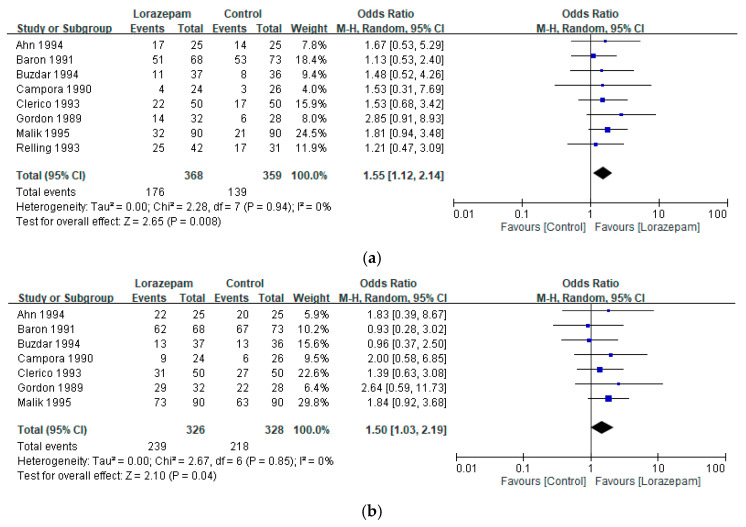
Pooled analysis of (**a**) complete response and (**b**) improved response for emesis with lorazepam-containing regimens versus comparator regimens in the prevention of chemotherapy-induced nausea and vomiting. Results are expressed as odds ratios (ORs) with 95% confidence intervals (CIs) using a random-effects model [11,21,22,23,24,25,26,27].

Pooled analysis has demonstrated that lorazepam-containing regimens were associated with a significantly higher complete response rate for emesis compared with comparator regimens (ORs: 1.55, 95% CI 1.12–2.14; I^2^ = 0%; *p* = 0.008) (Figure 3a). Similarly, the improved response rate for emesis was significantly greater in the lorazepam arms (ORs: 1.50, 95% CI 1.03–2.19; I^2^ = 0%; *p* = 0.04) (Figure 3b). Adjunctive lorazepam may provide additional benefits in controlling vomiting beyond those achieved with standard antiemetic therapy alone.

### 3.4. Overall Nausea Control

**Figure 4:**
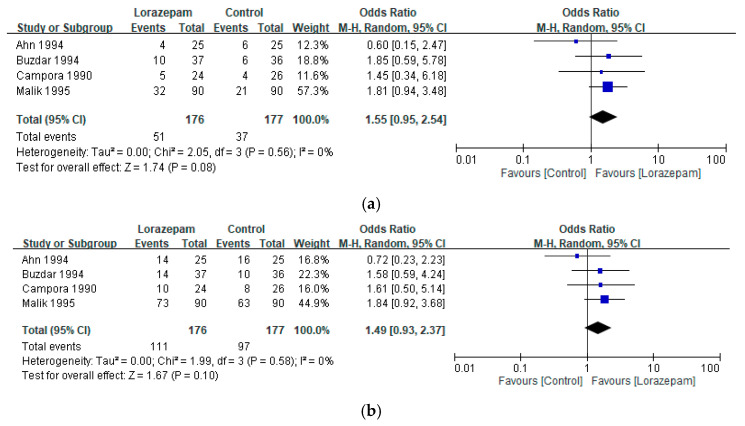
Pooled analysis of (**a**) complete response and (**b**) improved response for nausea with lorazepam-containing regimens versus comparator regimens in the prevention of chemotherapy-induced nausea and vomiting. Results are expressed as odds ratios (ORs) with 95% confidence intervals (CIs) using a random-effects model [11,21,23,24].

The results for nausea control indicated a positive trend favoring lorazepam, although it did not reach the threshold for statistical significance. The pooled ORs indicated higher rates of complete response (ORs: 1.55, 95% CI 0.95–2.54; I^2^ = 0%; *p* = 0.08) (Figure 4a) and improved response (ORs: 1.49, 95% CI 0.93–2.37; I^2^ = 0%; *p* = 0.10) (Figure 4b) compared with control regimens. These results highlight the efficacy of lorazepam in reducing both the incidence and severity of chemotherapy-related nausea, an outcome that remains challenging despite contemporary antiemetic prophylaxis.

### 3.5. Acute Phase Efficacy

**Figure 5:**
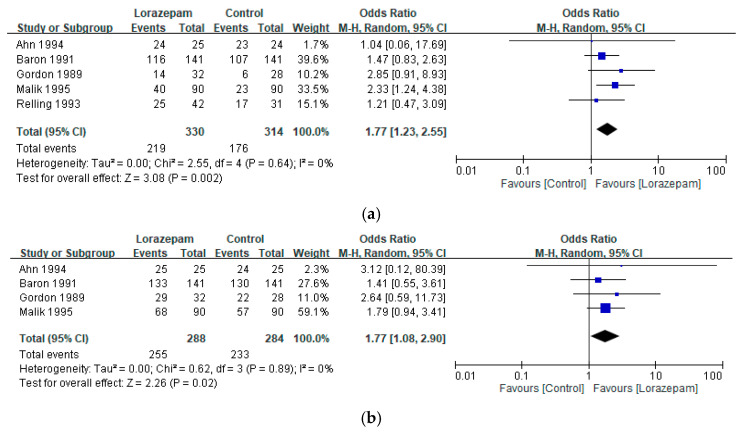
Pooled analysis of (**a**) complete response and (**b**) improved response for acute emesis with lorazepam-containing regimens versus comparator regimens in the prevention of chemotherapy-induced nausea and vomiting. Results are expressed as odds ratios (ORs) with 95% confidence intervals (CIs) using a random-effects model [11,21,22,26,27].

Subgroup analysis restricted to the acute phase (within 24 h of chemotherapy) revealed consistent benefits. Lorazepam-containing regimens significantly increased the likelihood of complete response for acute emesis (ORs: 1.77, 95% CI 1.23–2.55; I^2^ = 0%; *p* = 0.002) (Figure 5a) and of improved response for acute emesis (ORs: 1.77, 95% CI 1.08–2.90; I^2^ = 0%; *p* = 0.02) (Figure 5b) relative to comparators, indicating that lorazepam contributes meaningfully to early-phase emesis control.

### 3.6. Safety and Tolerability

**Figure 6:**
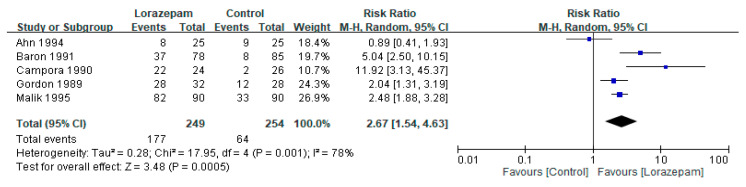
Pooled analysis of adverse events (sedation) with lorazepam-containing regimens versus comparator regimens in the prevention of chemotherapy-induced nausea and vomiting. Results are expressed as risk ratios with 95% confidence intervals (CIs) using a random-effects model [11,21,22,24,26].

Sedation was the most frequently reported adverse event. The pooled risk ratio showed a higher incidence of sedation with lorazepam-containing regimens compared with control arms (risk ratios: 2.67, 95% CI 1.54–4.63; I^2^ = 78%; *p* = 0.0005) (Figure 6). However, no serious lorazepam-related complications such as respiratory depression or severe hypotension were reported, and most sedation events were mild to moderate in intensity.

### 3.7. Sensitivity Analysis

Sensitivity analysis was performed using the leave-one-out approach to evaluate the robustness of the meta-analysis findings for complete response to emesis. The pooled ORs ranged from 1.47 to 1.60 across iterations, with corresponding 95% CIs ranging from 1.05 to 2.38 (Table 2). Heterogeneity remained consistently low (I^2^ = 0%), regardless of which study was excluded. The meta-analysis findings were generally robust and not driven by any single study.

### 3.8. Publication Bias

**Figure 7:**
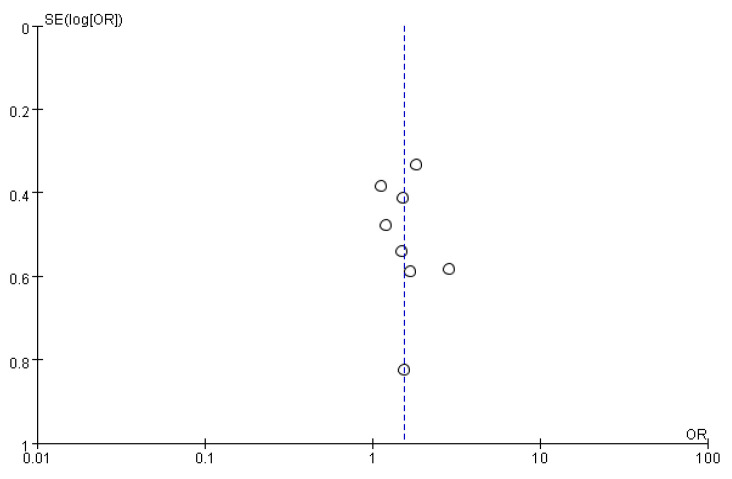
Funnel plot for the comparison of lorazepam-containing regimens versus comparator regimens for the outcome of complete response of emesis.

Visual inspection of the funnel plot for the primary outcome (complete response for emesis) revealed slight asymmetry (Figure 7). Publication bias was assessed using Begg’s and Egger’s tests. The Begg’s test showed a Kendall’s tau of −1.73 (*p* = 0.0833), and Egger’s test showed a regression intercept of −0.682 (standard error = 0.300; 95% CI: −1.418 to 0.054; *p* = 0.0633), indicating no statistically significant evidence of publication bias. Given that only eight studies were included, the reliability of these tests was limited, and the results should be interpreted with caution.

## 4. Discussion

This comprehensive systematic review and meta-analysis demonstrated that the addition of lorazepam to standard antiemetic regimens confers a clinically meaningful improvement in the control of chemotherapy-induced vomiting, particularly during the acute phase, and offers a modest but not statistically significant benefit for nausea prevention. The pooled estimates revealed significantly higher rates of complete and improved responses to vomiting with lorazepam-containing regimens than with control therapies, and the most frequent adverse event was sedation. Importantly, no serious adverse effects were reported, indicating the relative safety of lorazepam when appropriately monitored.

CINV can be categorized into three distinct phases: acute (0–24 h), delayed (24–72 h), and anticipatory (occurring before treatment and triggered by psychological and conditioned reflexes) [28]. Acute CINV is primarily mediated by serotonin release from enterochromaffin cells in the gastrointestinal tract. In contrast, delayed and anticipatory CINV involves the dopaminergic, neurokinin, and higher cortical centers associated with anxiety and conditioned responses. Standard antiemetic regimens, including 5-HT3 antagonists and corticosteroids, have significantly improved the control of acute and delayed CINV; however, some patients continue to experience difficult-to-manage symptoms, particularly anticipatory emesis.

Lorazepam, a benzodiazepine, likely exerts its effects through GABA-A receptor modulation in the central nervous system, reducing the cortical input and anxiety associated with chemotherapy. While this mechanism is highly effective in suppressing the vomiting reflex—particularly anticipatory emesis—nausea pathways involve more complex peripheral and chemoreceptor trigger zone (CTZ) interactions that may require synergistic inhibition from other agents like 5-HT3 or NK1 receptor antagonists. Recent interventional evidence supports this psychological mechanism; a 2017 study by James et al. demonstrated that lorazepam significantly reduced psychological distress, lowering Hamilton Anxiety (HAM-A) and Distress Thermometer (DT) scores, which subsequently improved the mental component of patients’ quality of life. Furthermore, they observed that when lorazepam was combined with contemporary agents such as aprepitant (an NK1 receptor antagonist), there was an 85% decrease in both nausea and vomiting, suggesting a synergistic effect between anxiolytic properties and modern receptor-specific antagonists [7].

Beyond mechanistic considerations, the included studies encompassed a diverse range of malignancies, including epithelial neoplasms and lymphoid neoplasms. Despite this histological diversity, our meta-analysis revealed remarkably low heterogeneity (I2 = 0%) across all primary outcomes, suggesting that the adjunctive benefit of lorazepam is likely independent of the tumor’s histological origin. However, results in specific modern cohorts may vary; for instance, Ono et al. (2019) [19] found no significant beneficial effect of adjunctive lorazepam when added to granisetron-based regimens in pediatric patients with acute lymphoblastic leukemia (ALL). This indicates that in certain lymphoid malignancies or specific age groups, other risk factors like female gender and older age may be more critical predictors of emetic control than the addition of lorazepam.

The present findings underscore the value of lorazepam as an adjunct to contemporary antiemetic therapy, particularly for acute-phase emesis. Although our findings indicate that sedation was more frequently observed in the lorazepam groups (RR = 2.67; 95% CI, 1.54–4.63), this effect should not be viewed solely as an adverse outcome. While these sedative properties may limit the functional recovery of outpatients, they may also confer therapeutic benefits for patients experiencing high levels of distress or insomnia during chemotherapy. Therefore, the use of adjunctive lorazepam should be individualized, with careful dose selection and monitoring being warranted to strike a balance between efficacy and tolerability, potentially prioritizing patients with prominent anxiety or those who have failed to achieve adequate control with standard antiemetic.

Clinically, lorazepam is recommended to start at doses of 0.5–2 mg the night before chemotherapy, with a repeat dose 1–2 h prior to treatment. It may be used as an adjunct to standard antiemetic therapy or, in select cases, as monotherapy when conventional regimens are ineffective [29]. Overall, these data support the integration of lorazepam into antiemetic regimens for patients at risk of acute or anticipatory emesis, particularly when standard therapy provides incomplete relief. Future studies should evaluate lorazepam in combination with current multidrug prophylaxis (e.g., NK1 antagonists or olanzapine) and identify patient subgroups, such as those with high baseline anxiety, who may derive the most significant benefit. Optimizing dosing schedules to minimize sedation while maintaining antiemetic efficacy remains a priority.

Several limitations of this review warrant consideration. First, most included trials were conducted in the 1980s and 1990s, prior to the routine implementation of contemporary antiemetic standards such as palonosetron or olanzapine. Consequently, the incremental benefit of lorazepam when added to current guideline-consistent therapy remains to be fully elucidated. Second, the small number of studies (*n* = 8) limited the power of our publication bias assessments. Despite these limitations, this study provides the most comprehensive synthesis of evidence to date, supporting lorazepam as a viable adjunctive option for emesis prophylaxis. Third, despite being double-blind RCTs, several trials had incomplete reporting of randomization, intervention details, or outcome assessment, introducing a potential risk of bias. Fourth, definitions and assessments of nausea and vomiting varied across studies, particularly for anticipatory symptoms, which were often subjectively evaluated. Fifth, although we observed consistent efficacy across various malignancies, the lack of granular data on specific histological subtypes, such as neuroglial neoplasms, in historical trials remains a limitation. Finally, safety data were primarily short-term and focused on sedation, with limited information on long-term or uncommon adverse effects. Therefore, caution is needed when applying these findings to contemporary clinical settings.

## 5. Conclusions

In addition to standard antiemetic therapy, this meta-analysis demonstrates that lorazepam significantly improves the complete response rate for chemotherapy-induced vomiting, particularly during the acute phase (OR = 1.77). While a favorable clinical trend was observed for nausea control, it did not reach statistical significance (*p* = 0.08). Sedation is the most common side effect (RR = 2.67); however, serious complications such as respiratory depression are rare. Despite the limitations of relying on historical trials, lorazepam remains a safe and beneficial adjunct for patients with symptoms that are difficult to control, such as anticipatory nausea and vomiting. The clinical application of lorazepam should be individualized, balancing its antiemetic and anxiolytic benefits against the increased risk of sedation. Future research should focus on its efficacy when combined with modern chemotherapy regimens and standardized outcome measures.

## Figures and Tables

**Table 1:** Overview of characteristics for included studies.

Study	Study Design	Chemotherapy/Cancer Types	Sample Size	Arms	Intervention	Outcome
Ahn 1994 [21]	Double blind RCT	Cisplatin-based/ Solid tumors, predominantly stomach/gastric cancer	75	Three-arm	1. Ondansetron 2. Ondansetron + Dexamethasone 3. Ondansetron + Dexamethasone + Lorazepam	Acute and delayed emesis
Baron 1991 [22]	RCT	Cisplatin–based and Non–Cisplatin–based/Various cancers	282	Two-arm	1. Metoclopramide + Methylprednisolone 2. Metoclopramide + Methylprednisolone + Lorazepam	Acute and delayed emesis
Buzdar 1994 [23]	Double blind RCT	Cyclophosphamide, Doxorubicin, Dacarbazine, Vincristine, Carmustin, Fluorouracil, Cisplatin/Breast cancer	94	Two-arm	1. Dexamethasone + Promethazine 2. Dexamethasone + Promethazine + Lorazepam	Delayed emesis
Campora 1990 [24]	Double blind RCT	Fluorouracil, doxorubicin, Cyclophosphamide, Methotrexate/Breast cancer	53	Two-arm	1. Methylprednisolone 2. Methylprednisolone + Lorazepam	Delayed emesis
Clerico 1993 [25]	Double blind RCT	Cisplatin-containing/Various cancers	60	Two-arm	1. Placebo 2. Lorazepam	Delayed emesis
Gordon 1989 [26]	Double blind RCT	Cisplatin–based/Various cancers	60	Two-arm	1. Methylprednisolone 2. Methylprednisolone + Lorazepam	Acute emesis
Malik 1995 [11]	Prospective randomized trial	High dose of Cisplatin/Various cancers	180	Two-arm	1. Metoclopramide + Dexamethasone 2. Metoclopramide + Dexamethasone + Lorazepam	Acute and delayed emesis
Relling 1993 [27]	Double blind RCT	Teniposide plus Cytarabine/Acute lymphoblastic leukemia	63	Two-arm	1. Chlorpromazine alone 2. Chlorpromazine + Lorazepam	Acute emesis

**Table 2:** Sensitivity analysis using the leave-one-out approach for lorazepam-containing regimens versus comparator regimens in the prevention.

Study	ORs	Lower CI	Upper CI	I^2^ (%)	*p*-Value
Overall	1.55	1.12	2.14	0	0.008
Omitting					
Ahn 1994 [21]	1.54	1.10	2.15	0	0.01
Baron 1991 [22]	1.66	1.16	2.38	0	0.005
Buzdar 1994 [23]	1.56	1.12	2.18	0	0.01
Campora 1990 [24]	1.55	1.11	2.15	0	0.009
Clerico 1993 [25]	1.55	1.09	2.21	0	0.01
Gordon 1989 [26]	1.47	1.05	2.06	0	0.03
Malik 1995 [11]	1.47	1.01	2.13	0	0.04
Relling 1993 [27]	1.60	1.13	2.26	0	0.007

## Data Availability

All data, models, and codes generated or used in the study appear in the submitted article.

## Supplementary Materials

The following supporting information can be downloaded at: https://www.mdpi.com/article/10.3390/biomedicines14040924/s1, Table S1. The PRISMA checklist.

## Abbreviations

The following abbreviations are used in this manuscript:

CINV	Chemotherapy-induced nausea and vomiting
QOL	Quality of life
5-HT3	5-hydroxytryptamine type 3
GABA	Gamma-aminobutyric acid
PRISMA	Preferred Reporting Items for Systematic Reviews and Meta-Analyses
CTZ	Chemoreceptor trigger zone

## Appendix A

PubMed (last searched August 2025)

Search: (“Lorazepam”[Mesh] OR lorazepam[tiab] OR ativan[tiab]) AND (“Nausea”[Mesh] OR “Vomiting”[Mesh] OR “Chemotherapy-Induced Nausea and Vomiting”[Mesh] OR nausea[tiab] OR vomiting[tiab] OR “chemotherapyinduced nausea”[tiab] OR “chemotherapy-induced vomiting”[tiab] OR “chemotherapy related nausea”[tiab] OR “chemotherapy related vomiting”[tiab] OR CINV[tiab]) AND (“Acute Disease”[Mesh] OR acute[tiab] OR “acute phase”[tiab]) AND (“randomized controlled trial”[pt] OR “controlled clinical trial”[pt] OR randomized[tiab] OR randomly[tiab] OR placebo[tiab] OR trial[tiab]) Filters: Randomized Controlled Trial Sort by: Most Recent N = 23

Embase (via Ovid, last searched August 2025)

1. exp lorazepam/OR lorazepam.ti,ab. OR ativan.ti,ab.
2. exp nausea/OR exp vomiting/
  OR nausea.ti,ab. OR vomiting.ti,ab.
  OR “chemotherapy induced nausea”.ti,ab.
  OR “chemotherapy induced vomiting”.ti,ab.
  OR “chemotherapy related nausea”.ti,ab.
  OR “chemotherapy related vomiting”.ti,ab.
  OR CINV.ti,ab.
3. exp acute disease/OR acute.ti,ab. OR “acute phase”.ti,ab.
4. 1 AND 2 AND 3
5. exp randomized controlled trial/OR exp controlled clinical trial/
  OR randomized.ti,ab. OR randomly.ti,ab.
  OR placebo.ti,ab. OR trial.ti,ab.
6. 4 AND 5
7. Article
  N = 130

Cochrane Library (CENTRAL, last searched August 2025)

(lorazepam OR ativan)

AND

(nausea OR vomiting OR “chemotherapy induced nausea” OR “chemotherapy induced vomiting” OR CINV)

AND

(acute OR “acute phase”)

N = 59
